# The impact of psychosis genome-wide associated ZNF804A variation on verbal fluency connectivity

**DOI:** 10.1016/j.jpsychires.2017.12.005

**Published:** 2018-03

**Authors:** Diogo Tecelão, Ana Mendes, Daniel Martins, Elvira Bramon, Timothea Toulopoulou, Eugenia Kravariti, Robin Murray, Diana Prata

**Affiliations:** a Instituto de Medicina Molecular, Faculdade de Medicina da Universidade de Lisboa, Lisbon, Portugal; b Department of Psychosis Studies, Institute of Psychiatry, Psychology & Neuroscience, King's College London, London, UK; c Mental Health Neurosciences Research Department, Division of Psychiatry, University College London, London, UK; d Department of Psychology, The University of Hong Kong, Hong Kong Special Administrative Region; e The State Key Laboratory of Brain and Cognitive Sciences, The University of Hong Kong, Hong Kong Special Administrative Region; f Department of Psychology, Bilkent University, Ankara, Turkey; g Department of Basic and Clinical Neuroscience, The Institute of Psychiatry, Psychology and Neuroscience, King's College London, UK; h Department of Psychology, Institute of Psychiatry, Psychology & Neuroscience King's College London, London, UK; i Department of Neuroimaging, Institute of Psychiatry, Psychology & Neuroscience, King's College London, London, UK; j Instituto Universitário de Lisboa (ISCTE-IUL), Cis-IUL, Lisbon, Portugal

**Keywords:** Neuroimaging genetics, Schizophrenia, Bipolar disorder, Psychosis, Genome-wide association, ZNF804A

## Abstract

Schizophrenia (SCZ) and bipolar disorder (BD) have high heritability. Genome-wide association studies (GWAS) have identified *ZNF804A* as a significant risk gene for both illnesses. A validation of this finding at the brain systems-level is imperative as there is still little understanding of how it heightens risk. Based in part on our recent findings of an effect on widespread decreased white matter microstructural fractional anisotropy (putatively a proxy of its integrity), particularly strong in SCZ, we asked whether the risk allele has a detrimental effect on regional brain activation and functional connectivity during a type of cognitive processing which is, together with its neural correlates, impaired in BD and SCZ: verbal fluency. Functional MRI and genotype data was collected from 80 healthy volunteers, and 54 SCZ and 40 BD patients. A standard multifactorial analysis of variance using statistical parametric mapping and significance correction of FWE p < 0.05 was used. We found the GWAS risk allele A was associated with decreased positive functional coupling between the left precentral gyrus/inferior frontal gyrus (i.e. the most highly recruited area for the task) and: 1) the left inferior frontal gyrus, and 2) the left posterior cingulate gyrus, encompassing the precuneus; both as a main effect across controls and psychosis patients. Such association of the risk allele with reduced functional connectivity (with no area where the opposite main effect was detected), converges with findings in other tasks, our previous finding of its widespread impact on brain white matter microstructure, and with the dysconnectivity hypothesis of SCZ.

## 1 Introduction

Schizophrenia (SCZ) and bipolar disorder (BD) have high heritability. Genome-wide association studies (GWAS) have robustly identified *ZNF804A* as a significant risk gene for both illnesses (Gurung and Prata, 2015), by virtue of it containing the rs1344706 polymorphism for which the adenine (A) allele was slightly more common in both patient groups. A validation of this finding at the brain systems-level is imperative as there is still little understanding of how it heightens risk. *ZNF804A* expresses the zinc-finger protein 804A with a domain typical of a transcription factor in the developing hippocampus, the cortex, and the adult cerebellum (Gurung and Prata, 2015). Consistent with a neurodevelopmental role, we recently found the risk allele (A) to be highly significantly associated with widespread decreased white matter microstructural fractional anisotropy (putatively a proxy of its integrity) across healthy subjects, as well as patients with SCZ and with BD - but particularly strongly in SCZ (Mallas et al., 2016). We now asked whether it would, consequently, have a detrimental effect (and possibly larger in SCZ patients) on regional brain activation and functional connectivity during a type of cognitive processing which is, together with its neural correlates, affected in BD and SCZ, and their healthy relatives (Curtis et al., 2001, Daban et al., 2006): verbal fluency (Prata et al., 2009). Such effect would be consistent with previous findings associating it with a decrease in functional connectivity during working memory (Esslinger et al., 2009, Rasetti, 2011). On behaviour, effects seem smaller or less detectable, with one study showing a weak-to-modestly linked allele (in terms of linkage disequilibrium) to be marginally associated with better category fluency, but not verbal fluency, in healthy males (Nicodemus et al., 2014); and the present risk allele with worse visuo-motor performance, but again not verbal fluency nor verbal learning (Lencz et al., 2010).

## 2 Material and methods

We genotyped (for *ZNF804A* rs1344706) and scanned, with functional MRI, 174 English native speakers [80 healthy volunteers without history of mental illness, 54 patients with SCZ and 40 with BD (75% of whom had a history of psychosis symptoms)], recruited from the SLAM NHS Trust and diagnosed according to DSM-IV (methodological detail and references are available in Supplement 1). This yielded 84 rs134470684 allele A homozygotes and 90 allele C carriers, and given the very low frequency of allele C in Caucasian population, we grouped the non-risk allele C homozygotes with the heterozygotes. The study was approved and reviewed by the National Health Service (NHS) South East London Research Ethics Committee, UK (Project “Genetics and Psychosis (GAP)” reference number 047/04) and was carried in accordance with the latest version of the Declaration of Helsinki. Informed consent of the participants was obtained after the nature of the procedures had been fully explained. There were no demographic differences (at p < 0.05) between genotype or diagnostic groups, other than CPZ-equivalent medication and male:female being higher, and IQ lower, in patients with SCZ (see sample's demographics in Supplement 2). The verbal fluency task [where subjects are required to overtly generate a word starting with a visually-displayed letter; or overtly read the word “rest” (control or repetition condition)], image acquisition, pre-processing and analysis using SPM software was performed as described earlier (Prata et al., 2009). A multifactorial ANOVA was used to test for the main effect of genotype (A homozygotes vs. C carriers) and its interaction with diagnosis (controls vs. BD vs. SCZ; or all psychosis patients, i.e. 100% of SCZ group + 75% of BD group, vs. controls). These effects were tested on whole-brain regional activation and on functional connectivity, including connectivity that would depend on task trials (i.e. psychophysiological interaction; PPI). Main effect of task is reported for completeness, but not discussed, as it has already been described in a highly overlapping sample (Prata et al., 2009). Incorrect response trials were excluded to avoid confounding effects of task performance. For connectivity, we used, as seed, the 6-mm radius sphere where the main effect of task was the highest (i.e. left precentral gyrus/inferior frontal gyrus, pars opercularis: −44 4 34). η_p_^2^ measures of effect size were calculated in R to assess how much of the inter-individual (+error) variance in peak activation was explained by genotype.

## 3 Results and discussion

### 3.1 Effect of verbal fluency on regional activation

Consistently with our previous findings with overlapping samples (Prata et al., 2009), word generation (irrespective of task difficulty) was associated (whole-brain, voxel-wise FWE-corrected <0.05) with activation in an extended network that included the left inferior and middle frontal gyrus, middle and posterior cingulate gyrus, as well as the anterior cerebellum (see Supplementary Table 2 for further detail). Conversely, repetition was associated with activation bilaterally in the angular gyrus, anterior cingulate and middle occipital gyri, and rolandic operculum.

### 3.2 Effect of *ZNF804A* rs1344706 on regional activation

**Fig. 1:**
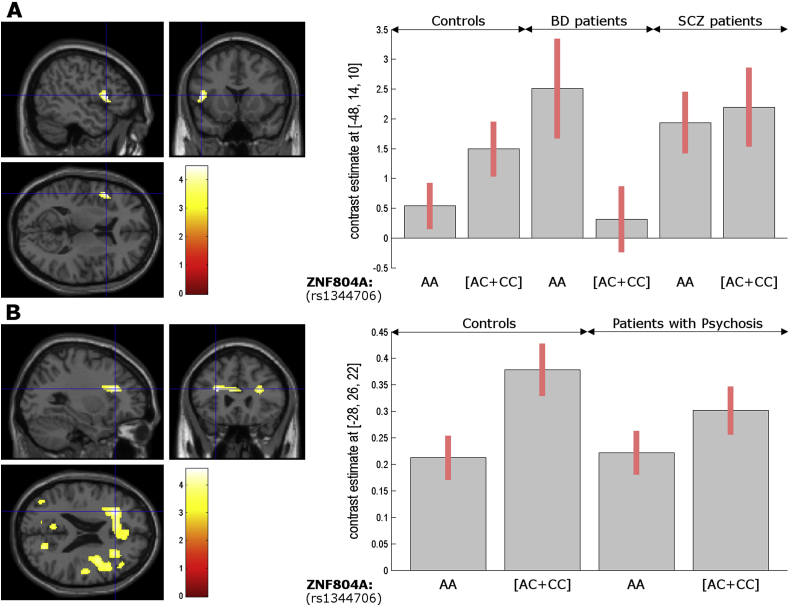
**– Part A:** Genotype-by-diagnosis interaction in the left inferior frontal gyrus, pars triangularis/opercularis, where risk allele adenine (A) [vs. Cytosine (C) carriers] was associated with higher regional activation (Y-axis) in BD, but the reverse was seen in healthy volunteers (X-axis). **Part B:** Main effect of *ZNF804A* rs1344706 genotype on functional connectivity, whereby risk allele A homozygotes [vs. Cytosine (C) carriers] showed decreased connectivity between the left precentral gyrus/inferior frontal gyrus, pars opercularis (seed) and the left inferior frontal gyrus, pars triangularis/opercularis (Y-axis), across controls and patients with history of psychosis (i.e. the whole SCZ and 75% of the BD group; X-axis). Regions are represented when surviving p < 0.001 uncorrected (see Table 1). Parameter estimates are for verbal fluency > repetition in the left inferior frontal gyrus, pars triangularis/opercularis (Part A: −48 14 10, z-score = 4.39, voxel-wise FWE-corrected p-value = 0.03; Part B: −28 26 22, z-score = 4.42, voxel-wise FWE-corrected p-value = 0.02; between-subjects SEM bars are in red). Brain regions are labelled using an automatic-labelling atlas^(∗)^ and confirmatory visual inspection of a manual book atlas^(∗∗)^. (*) Tzourio-Mazoyer, N., Landeau, B., Papathanassiou, D., Crivello, F., Etard, O., Delcroix, N., Mazoyer, B., Joliot, M., 2002. Automated Anatomical Labeling of Activations in SPM Using a Macroscopic Anatomical Parcellation of the MNI MRI Single-Subject Brain. NeuroImage 15, 273–289. https://doi.org/10.1006/nimg.2001.0978. (**) K. Mai, J., Paxinos, G., Voss, T., 2008. Atlas of the Human Brain, 3rd Edition, 3rd ed. Academic Press, San Diego. (For interpretation of the references to colour in this figure legend, the reader is referred to the Web version of this article.)

We found a genotype-by-diagnosis interaction in the left inferior frontal gyrus, pars opercularis/triangularis [Z = 4.39; whole-brain, voxel-wise FWE-corrected p = 0.03; η_p_^2^ (peak) = 4.32%; Fig. 1 - Part A], where risk allele A was associated with higher regional activation in BD, but the reverse was seen in healthy volunteers. This is indeed the area showing the highest activation in verbal fluency (vs. word repetition), and has been frequently attributed to phonological processing (Fiez, 1997). The latter genotype effect in healthy volunteers has been independently found in the same area during theory-of-mind (Walter et al., 2011). A report in BD, or verbal fluency for that matter, is unprecedented.

### 3.3 Effects of *ZNF804A* rs1344706 on functional connectivity

In terms of connectivity, we found the risk allele A was associated with decreased positive coupling between the left precentral gyrus/inferior frontal gyrus, pars opercularis seed (i.e. the most highly recruited area for the task) and: 1) the same region abovementioned under a genotype-by-diagnosis interaction [Z = 4.42; voxel-wise FWE-corrected p = 0.02 at a nearby white matter coordinate; η_p_^2^(peak) = 4.33%; Fig. 1 - Part B]; and 2) the left posterior cingulate gyrus [Z = 4.34; voxel-wise FWE-corrected p = 0.03; η_p_^2^(peak) = 7.60%], encompassing the precuneus; both as a main effect across controls and psychosis patients (Table 1). The latter effect in both areas was also present at FWE-corrected level when we only included SCZ and controls (and not present in BD alone at all, neither when we included the full BD group to the remaining groups) showing that these groups drove this main effect. In neither ANOVA models (with or without non-psychotic BD), did we find significant interaction effects. (Although unexpected, we note that white matter BOLD signals in a wide-variety of brain areas, task - or even resting-state-based fMRI have been extensively reported (Gawryluk et al., 2014) – and we have reported lower white matter fractional anisotropy, a putative proxy of integrity, to be widely associated with this polymorphism (Mallas et al., 2016)). Importantly, we found no area (even at a trend level of uncorrected p < 0.001) that showed to be under the opposite *main* effect (i.e. higher functional connectivity correlated with the risk allele A). Like the present findings in verbal fluency, studies have shown intra- (and also inter-) hemispheric prefrontal connectivity *decrease* (Esslinger et al., 2009), in working memory, facial emotion recognition and resting state (Esslinger et al., 2011) – which was replicated by others (Rasetti, 2011), albeit not always (Gurung and Prata, 2015). (Interestingly, in SCZ, the risk allele has been associated with increased functional connectivity *within* the right prefrontal cortex (Rasetti, 2011), as opposed to the abovementioned literature in health (Esslinger et al., 2009) – alerting to putative genotype-by-diagnosis interactions.) In fact, decreased prefrontal intra- and inter-hemispheric connectivity associated with this (SCZ) risk allele is consistent with the dysconnectivity hypothesis of SCZ, which has been especially supported between the hemispheres (Gurung and Prata, 2015). Another main finding in the literature is an association of the risk allele, during working memory, with *increased* functional connectivity between the right dorsolateral prefrontal cortex and the left *hippocampal* formation in health (Esslinger et al., 2009). (This has been replicated in Han Chinese (Zhang et al., 2016) and as a trend in another study (Gurung and Prata, 2015) but not by another report of *decreased* coupling (Rasetti, 2011) and yet another of decreased coupling between left hippocampal formation, this time with the posterior cingulate cortex (Zhang et al., 2016). This prefrontal-hippocampal effect was concluded to be task-specific since it was not verified in facial emotion recognition or resting state tasks in the same sample (Esslinger et al., 2011) - which likely explains why we have not replicated it in verbal fluency. In terms of PPI connectivity, we found no statistically significant (FWE p < 0.05) main effect of genotype or genotype-by-diagnosis interactions.

**Table 1:** **–** Regions showing an effect of *ZNF804A* rs1344706 genotype. All inferences presented in this table correspond to statistically significant results (corrected for whole-brain multiple comparisons, voxel-level FWE, p < 0.05) and trends (with an uncorrected p < 0.001) for the same effect. *P-values* (FWE *corrected*) *and cluster size* (*k*) *are given only for the areas showing statistically significant effects.*

Contrasts	Regions	Coordinates (x y z)	Z-score (Z), voxel-wise FWE corrected p-value (p), cluster size (k)
**1. Regional activations**			

***ZNF804A*****genotype-by-diagnosis interaction**			

(AA > AC + CC) & (BD > CON)	L Inferior frontal gyrus, pars triangularis/opercularis	−48 14 10	Z = 4.39, p = 0.026, k = 4
	L Insula	−42 14 8	Z = 3.13
**2. Functional Connectivity with Left Precentral gyrus/inferior frontal gyrus (peak of the main effect of task)**			
**Main effect of *ZNF804A* genotype**			
AA < AC + CC a	White matter near L Inferior frontal gyrus, pars triangularis/opercularis	−28 26 22	Z = 4.42, p = 0.021, k = 9
	L Posterior cingulate gyrus	−8 −52 32	Z = 4.34, p = 0.029, k = 11
	Left Precuneus	−6 −54 30	Z = 4.27, p = 0.04 (same cluster as above)
		−8 −50 34	Z = 4.25, p = 0.04 (same cluster as above)
	R Lingual gyrus	14 −84 −4	Z = 3.25
	L Middle occipital gyrus	−36 −82 30	Z = 3.46
		−42 −76 26	Z = 3.48
	L Angular gyrus	−44 −76 30	Z = 3.34
	R Cuneus	20 −68 22	Z = 3.89
	R Superior Occipital gyrus	22 −68 28	Z = 3.18
	R Precuneus	20 −66 22	Z = 3.78
		4 −58 32	Z = 3.88
	L Cuneus	−6 −66 28	Z = 3.37
	L Lingual gyrus	−8 −64 6	Z = 3.2
	R Calcarine sulcus (occipital gyrus)	20 −62 12	Z = 3.44
	L Calcarine sulcus (occipital gyrus)	−12 −62 12	Z = 3.84
	R Posterior Cingulate gyrus	4 −58 30	Z = 3.86
	R Middle Cingulate gyrus	2 −54 32	Z = 3.59
	R Angular gyrus	52 −52 34	Z = 3.25
	Anterior Cerebellum (Vermis IV/V)	2 −46 −12	Z = 3.16
	Anterior Cerebellum (Vermis III)	0 −40 −14	Z = 3.77
	L Middle cingulate gyrus	0 −36 34	Z = 4.14
	R Hippocampus	18 −34 8	Z = 3.85
		42 −18 −18	Z = 3.55
	R Superior temporal gyrus	44 −32 8	Z = 3.80
		54 −30 8	Z = 4.04
	R Thalamus	18 −32 8	Z = 3.71
	R Insula	36 −18 8	Z = 3.32
		38 3 16	Z = 3.14
	R Fusiform gyrus	42 −18 −20	Z = 3.54
	R Rolandic operculum	54 −16 10	Z = 3.43
		58 4 10	Z = 3.68
	R Transverse temporal gyrus	52 −16 8	Z = 3.52
		60 −2 6	Z = 3.2
	R Postcentral gyrus	50 −16 26	Z = 3.74
		52 −14 26	Z = 3.80
	R Putamen	34 −10 4	Z = 3.23
	R Precentral gyrus	44 2 24	Z = 3.29
	R Inferior frontal gyrus, pars opercularis	38 4 22	Z = 3.97
	L Caudate nucleus/Septal nuclei	0 6 4	Z = 3.38
	White matter near L Inferior frontal gyrus, pars opercularis	−34 8 24	Z = 3.2
	R Inferior frontal gyrus, pars triangularis	46 18 20	Z = 3.76
		48 32 20	Z = 3.46
	R Middle frontal gyrus	30 26 22	Z = 3.94
		46 34 20	Z = 3.42
	L Superior frontal gyrus, medial	−28 26 22	Z = 3.41
	White matter near L Anterior cingulate gyrus	−6 28 20	Z = 4.01
		−16 28 22	Z = 3.90
	L Middle frontal gyrus	−26 32 24	Z = 3.58
	R Anterior cingulate gyrus	2 32 18	Z = 3.53

AA, adenine-adenine; AC, adenine-cytosine; CC – cytosine-cytosine; CON, controls, BD, bipolar disorder; SCZ, schizophrenia; PSYCH, psychosis patients; R, right; L, left.

a Only present in the ANOVA comprising controls and patients with psychosis.

Moreover, these findings lend support to, and, in fact, are likely explained by, our recent results in an overlapping sample, of a widespread and significant decrease in microstructural white matter fractional anisotropy, a putative proxy of white matter integrity, via diffusion tensor MRI, associated to this same risk variant (allele A) (Mallas et al., 2016). Importantly, nowhere in the brain (at a trend level) did we find evidence for an association of this risk allele with the opposite effect we report in that study, i.e. of *higher* microstructural white matter integrity (Mallas et al., 2016). We thus provide evidence that supports the dysconnectivity hypothesis of SCZ (Stephan et al., 2006), and a contributing role of rs1344706 risk allele, and extend its impact from the previously reported in working memory and emotional paradigms, to verbal fluency for the first time.

### 3.4 Potential confounding factors

As none of our demographic or medication variables correlated with genotype, they are unlikely to be confounding variables. In addition, we found no variable to affect brain activation in areas that we report to be under a genotype effect. We also found no relevant change in effect size or foci of activation of genotype effects when these variables were introduced in the ANOVA. Thirdly, no variable correlated with the peak activation values retrieved from our genotype effect analyses.

### 3.5 Complementary gene expression analyses

For complementary information, we searched a publicly available online database to determine whether the risk allele (or, rather, the linkage disequilibrium block it tags) affected ZNF804A mRNA expression level in any of the available 10 brain areas (i.e. was a cis-eQTL) (Ramasamy et al., 2014). We found the rs1344706 risk allele to be associated (at p < 0.05, uncorrected for brain areas) with increased ZNF804A exonic transcription levels in the hippocampus, medulla oblongata and occipital cortex (details in Supplement 4). For completeness, we also provide an analysis of Allen Brain Atlas data to define maps of ZNF804A expression in the human brain: relatively ZNF804A-enriched areas (mean normalized Z-score >1; details in Supplement 5) were the bed nucleus of stria terminalis, the nucleus of the diagonal band, the olfactory tubercle, the septal nuclei, the claustrum, the medial habenula, the hippocampal formation, the hypothalamus and the oculomotor and cochlear nuclei. Of note, from both these analyses the hippocampus was one of the areas where we found, at uncorrected p < 0.001, a main effect of genotype on functional connectivity with the seed (of the main effect of task) during verbal fluency.

## 4 Conclusion

In summary, we report statistically significant associations between genome-wide psychosis-implicated *ZNF804A* rs1344706 risk allele in a genotype-by-BD-diagnosis interaction on regional activation, and across psychosis patients and healthy samples with decreased functional connectivity, during verbal fluency – a task engaging brain regions and cognitive processes impaired in SCZ and BD. This converges with findings in other tasks (Gurung and Prata, 2015), our previous finding of its widespread impact on brain white matter microstructure (Mallas et al., 2016), and with the dysconnectivity hypothesis of SCZ.

## Appendix A Supplementary data

Supplement 5 Supplement 5

Data Profile Data Profile
