# Supplementary material for: The impact of psychosis genome-wide associated ZNF804A variation on verbal fluency connectivity
Source: J Psychiatr Res. 2018 Mar;98:17–21. doi: 10.1016/j.jpsychires.2017.12.005 (PMC5793999; doi:10.1016/j.jpsychires.2017.12.005)
Supplement: Supplement 1 [file mmc1.docx]

***SUPPLEMENT 1***

***Supplementary Methods***

***Subjects assessment.*** Our sample consisted of 174 English native speakers including a control group comprised of 80 healthy volunteers (34 males, 39±13 y.o.) with no history, or first degree family history, of a psychotic spectrum disorder, 54 patients with established SCZ (42 males, 37±11 y.o.) and 40 with BD (16 males, 40±12 y.o., 75% of which with psychosis). Patients were recruited from the South London and Maudsley (SLaM) NHS Trust. Diagnosis, according to the criteria of the Diagnostic and Statistical Manual of Mental Disorders (DSM) 4^th^ Edition^1^, was ascertained by an experienced psychiatrist using a structured diagnostic interview with instruments detailed elsewhere^2^. All SCZ and BD patients were in a stable clinical state. Exclusion criteria applied to all participants were a history of significant head injury and current (last 12 months) substance dependency according to DSM-IV diagnostic criteria. The study was approved by the National Health Service (NHS) South East London Research Ethics Committee, UK (Project “Genetics and Psychosis (GAP)” reference number 047/04). All subjects gave written informed consent in accordance.

***Genotyping.*** Genotyping for the *ZNF804A* single nucleotide polymorphism (SNP) rs1344706 was performed using standard genotyping techniques we previously described^3^. Possible genotype outcomes were A homozygous (AA, adenine-adenine), heterozygous (AC, adenine-cytosine) or C homozygous (CC, cytosine-cytosine). Given the very low frequency of allele C in Caucasian population, we grouped the non-risk allele C homozygotes with the heterozygotes. The grouping of the non-risk allele homozygotes (CC) with the heterozygotes (AC) was performed given the low frequency of the C allele (which is reflected on the Supplementary Table 1). Examples of this design can be found in several other studies testing the effect of the ZNF804A rs1344706 SNP we have previously systematically reviewed^4^.

The distribution of Caucasian genotype frequencies for the *ZNF804A* (0.46 AA, 0.39 AC, 0.15 CC) was consistent with Hardy-Weinberg Equilibrium, calculated using Michael H. Court calculator^5^, in patients (χ^2^ =1.60, df=1, p-value=0.21) and controls (χ^2^ =1.07, df=1, p-value=0.30).

***Verbal Fluency task*.** During a “generation” condition subjects were presented with a series of letters on a computer screen and were required to overtly articulate a word beginning with each presented letter. This was contrasted with a “repetition” condition in which subjects were presented with the word “rest” and were required to say “rest” out loud. A blocked design was used, with letter and “rest” cues presented in blocks of seven. The demands of the generation condition were manipulated experimentally by presenting different sets of letters that have previously been found to make the task relatively “easy” or “hard”^6^. Functional MRI data were acquired when five blocks of “rest” trials alternated with five blocks of “easy” letters (“easy” run) or of “hard” letters (“hard” run). This resulted in a total of 70 letter stimuli (35 easy, 35 hard) and 70 “rest” trials for each subject. The order of the “easy” and “hard” runs was counter-balanced across subjects. Verbal responses were recorded by means of a microphone that was compatible with the MRI apparatus; this allowed us to identify "incorrect" trials in which the subject did not generate any response or generated repetitions, derivatives or grammatical variations of a previous word.

**Image Acquisition**. T_2_*-weighted gradient-echo single-shot echo-planar images were acquired on a 1.5-T, neuro-optimized IGE LX System (General Electric, Milwaukee) at the Maudsley Hospital, London, U.K. Twelve noncontiguous axial planes (7-mm thickness, slice skip: 1 mm, 3.75 x 3.75 mm voxel size in plane, and 64 x 64 mm matrix size in plane) parallel to the anterior commissure-posterior commissure line were collected over 1100ms in a "clustered" acquisition (TE=40 ms, flip angle=70°) which permitted articulatory responses to be made when images were not being acquired, minimising the effects of head movement on the BOLD signal^6^. Immediately after each acquisition a letter was presented (remaining visible for 750 ms, height: 7 cm, subtending a 0.4° field of view), and a single overt verbal response was made during the remaining silent portion (entire duration=2900ms) of each repetition (TR=4000ms), with an image acquired during 1100 ms. Head movement was minimized by a forehead strap. To ensure that subjects heard their responses clearly, their speech was amplified by a computer sound card and then relayed to the subject through an acoustic MRI sound system and noise-insulated, stereo headphones.

***Neuroimaging data preprocessing and analysis.*** Data preprocessing was performed using SPM8 software (University College London, UK) running under Matlab 8.3 (The Mathworks, Inc., USA). All volumes from each subject were realigned and unwarped (using the first slice as reference), with a separation of 4mm between the points sampled in the reference image, a 5 mm full width at half maximum (FWHM) isotropic Gaussian kernel applied to the images before estimating the realignment parameters, and 2^nd^ degree B-spline interpolation. Normalization to the functional MNI template (EPI) was then performed using a voxel size of 2x2x2mm and trilinear interpolation. Spatial smoothing was carried out with an 8 mm FWHM isotropic Gaussian kernel. The remaining realignment, unwarping, normalization and smoothing options corresponded to the default choices in SPM8.

After the pre-processing steps, statistical analysis of regional responses in a subject-specific fashion was performed using SPM12, by convolving each onset time with a synthetic haemodynamic response function (HRF)^7^. The application of an event-related model resulted in five experimental conditions: 1) easy; 2) repetition-easy; 3) hard; 4) repetition-hard; 5) wrong. The latter was excluded from the group analysis so we could control for effects of group differences in task performance (and as such limit our inferences to scans corresponding to correct responses). Data were high-passed filtered with a cut-off period of 128s using a set of discrete cosine basis function. Parameter estimates were calculated for all brain voxels recurring to a general linear model, and contrast images for “verbal fluency (easy+hard) > repetition” were created for each subject independently. This allowed the estimation of the main effect of task. Between-subject inferences were obtained entering the subject-specific contrast images for “verbal fluency (easy+hard) > repetition” into a 3 x 2 full-factorial ANOVA (‘Diagnosis’ x ‘ZNF804A genotype’). [A complementary secondary analysis was performed where the levels of ‘Diagnosis’ were ‘healthy volunteers’ and ‘patients with psychosis’ (i.e. all SCZ plus 75% of the BD patients)]. Since the superior region of the prefrontal cortex was not scanned in a sub-group of subjects, it was automatically excluded from the 2nd level analyses. We tested for the main effect of ZNF804A genotype and for its interaction with diagnosis, on whole-brain regional activation and, as described below, on functional connectivity.

***Functional Connectivity and Psychophysiological Interactions***. Apart from direct effects of individual regions’ activity, we aimed to know if the genetic variations also affected functional connectivity, inclusive connectivity that is necessarily dependent on the task paradigm (psychophysiological interactions; PPI)^8^. For that, we used the same ANOVA model as above this time using (instead of activation) coupling (i.e. time-correlated activation) between the (seed) 6-mm radius sphere ROI where the main effect of task was the highest (i.e. left precentral gyrus/inferior frontal gyrus, pars opercularis, tagged by its peak coordinates: -44 4 34) and the remaining brain. Using the same seed, PPI analyses were also conducted to find brain areas in which there were variations in functional connectivity dependent on the task design (i.e. that would be significantly different during verbal fluency trials and ‘repetition’ trials). This was performed by doing a vector multiplication of the seed time series with the HRF convolved task (using “VF (easy+hard) > repetition” contrasts). The resulting PPI vector was then used as a regressor in a subject-level analysis, with both the seed time series and the HRF convolved task as covariates of no interest. Thereafter, the resulting subject-specific contrast images were entered into the same ANOVA design as above to make inferences at the population level.

***Results’ effect size.*** To assess how much of the interindividual (+ error) variance in blood oxygen level-dependent activation was explained by variation in genotype, we used the η_p_^2^ (partial eta squared) measure of effect size in R software^9^, after extracting the subjects’ beta-measure at the voxel of peak activation.

***Potentially confounding factors in neuroimaging data analysis.*** To ascertain that none of our extraneous (demographic, psychopathological or medication) variables confounded, or added significant noise to our ANOVA results (only IQ and gender were significantly different between diagnoses group, and no extraneous variable was correlated with genotype; Supplementary Table 1), we performed extra analyses: 1) we included gender, age, handedness, ethnicity, IQ, years of education, duration of illness, CPZ, antipsychotic medication generation and duration (separately and together) in the ANOVA as covariates of no interest to check if they changed (more than 0.5) any Z- or T-statistic or the foci of maximal effect; 2) in a simple linear regression design, we examined whether each variable correlated (more significantly than p=0.01, uncorrected, and with a search radius of 6mm) with brain activation in any area overlapping with those found to be under significant effect after our ANOVA; and 3) we extracted the beta activation values at the peak (most significant) voxel from our ANOVA and separately asked if it was correlated with each demographic variable.

As none of our demographic or medication variables correlated with genotype, they are unlikely to be confounding variables. In addition, we found no variable to affect brain activation in areas that we report to be under a genotype effect. We also found no relevant change in effect size or foci of activation of genotype effects when these variables were introduced in the ANOVA. Thirdly, no variable correlated with the peak activation values retrieved from our genotype effect analyses.

***Complementary gene expression report*.** To investigate whether the rs1344706 variant (or other variants tagged by it in the same linkage disequilibrium block) affected ZNF804A mRNA expression level (i.e. was an eQTL), we searched the publicly available Braineac database - which includes genotypic and microarray profiling of 10 brain regions of 134 neuropathologically normal individuals with European descent^10^ (cerebellar cortex, frontal cortex, hippocampus, medulla oblongata, occipital cortex, putamen, substantia nigra, temporal cortex, thalamus, and intralobular white matter). We retrieved p-values for the association between this SNP genotype and the levels of expression for ZNF804A’s exon-specific probes and total transcript (Winsorised mean over exon-specific levels). More detailed information is described in the Braineac database^10^. Data were extracted and summarized on supplement 5.

***Complementary regional map of ZNF804A distribution in the human brain*:** To gain insight about the putative distribution of ZNF804A in the human brain, we took advantage of the transcriptomic data available in the Allen Brain Atlas (ABA) depository to explore the regional distribution of its mRNA and identify areas enriched in its expression. The ABA is open-source depository of transcriptomic data which characterizes gene expression in human brain tissue with genome-wide microarray-based gene expression profiles including over 62 000 gene probes for 500 samples from each hemisphere covering the whole brain. Microarray analysis data, normalized across each individual brain, are included in the Allen Human Brain Atlas data set and illustrated in heat map format as *Z* scores or log2 expression. *Z* scores represent individual regional gene expression normalized to the whole brain expression of that gene. To date (search conducted on the 22 May 2017), 6 subjects without a history of neuropsychiatric or neurological conditions are contained in the database. Detailed information for subjects included and analysis methods is available at [www.brain-map.org](http://www.brain-map.org/). Note that these subjects were not included in our imaging study. Gene expression of ZNF804A was analyzed in one probe in the Allen Human Brain Atlas - A_32_P316136. To calculate mean values of normalized expression in the brain structures available we extracted normalized *Z* scores from the database for each individual and structure. Since information for structures of the right hemisphere is only available for 2 individuals, we decided to focus our analysis on the left hemisphere (available for the whole sample). We also excluded all the structures not containing data of more than 3 individuals. Detailed information about the regional distribution of ZNF804A in the human brain can be consulted in Supplement 5.

**References**

1 American Psychiatric Association. *Diagnostic and statistical manual of mental disorders: DSM-IV*. American Psychiatric Association, 1994.

2 Prata DP, Mechelli A, Picchioni MM, Fu CH, Toulopoulou T, Bramon E, *et al.* Altered effect of dopamine transporter 3′ UTR VNTR genotype on prefrontal and striatal function in schizophrenia. *Arch Gen Psychiatry* 2009; **66**: 1162–1172.

3 Mallas E-J, Carletti F, Chaddock CA, Woolley J, Picchioni MM, Shergill SS, *et al.* Genome-wide discovered psychosis-risk gene ZNF804A impacts on white matter microstructure in health, schizophrenia and bipolar disorder. *PeerJ* 2016; **4**: e1570.

4 Gurung R, Prata DP. What is the impact of genome-wide supported risk variants for schizophrenia and bipolar disorder on brain structure and function? A systematic review. *Psychol Med* 2015; **45**: 2461–80.

5 Court MH. 2005-2008. Court’s online calculator. Tuft University Web site. (http://www.tufts.edu/~mcourt01/Documents/Court%20lab%20-%20HW%20calculator.xls).

6 Fu CHY, Morgan K, Suckling J, Williams SCR, Andrew C, Vythelingum GN, *et al.* A Functional Magnetic Resonance Imaging Study of Overt Letter Verbal Fluency Using a Clustered Acquisition Sequence: Greater Anterior Cingulate Activation with Increased Task Demand. *NeuroImage* 2002; **17**: 871–9.

7 Mechelli A, Prata DP, Fu CHY, Picchioni M, Kane F, Kalidindi S, *et al.* The effects of neuregulin1 on brain function in controls and patients with schizophrenia and bipolar disorder. *NeuroImage* 2008; **42**: 817–26.

8 Friston KJ, Buechel C, Fink GR, Morris J, Rolls E, Dolan RJ. Psychophysiological and Modulatory Interactions in Neuroimaging. *NeuroImage* 1997; **6**: 218–29.

9 R Core Team. *R: A language and environment for statistical computing*. R Foundation for Statistical Computing, 2016 (https://www.R-project.org/).

10 Ramasamy A, Trabzuni D, Guelfi S, Varghese V, Smith C, Walker R, *et al.* Genetic variability in the regulation of gene expression in ten regions of the human brain. *Nat Neurosci* 2014; **17**: 1418–28.
