# Supplementary material for: The impact of psychosis genome-wide associated ZNF804A variation on verbal fluency connectivity
Source: J Psychiatr Res. 2018 Mar;98:17–21. doi: 10.1016/j.jpsychires.2017.12.005 (PMC5793999; doi:10.1016/j.jpsychires.2017.12.005)
Supplement: Supplement 2 [file mmc2.docx]

***SUPPLEMENT 2***

Demographic differences between diagnostic and/or genotype groups were analysed using the R software^1^ recurring to chi-square tests (for categorical variables), Levene’s tests of equality of variances, independent t-tests and analysis of variance (ANOVA; for continuous variables). There were no significant differences in age, years of education, ethnicity or handedness between the groups of diagnosis, genotypes or genotypes in each diagnosis. IQ significantly differed (p<0.001) between diagnoses, being significantly lower in SCZ compared to controls (or BD) – but there were no significant differences in IQ between genotype groups (of either gene). Diagnoses also significantly (p<0.001) differed in gender with more males in SCZ than in BD and more females in controls than in SCZ. The patient groups also differed in chlorpromazine (CPZ) equivalents in medication (p<0.001) with SCZ having a higher load than BD.

**Supplementary Table 1 –** Participant's demographics per diagnosis and genotype groups.

|  | **Diagnosis** | | | | **ZNF804A rs1344706 Genotype** | | |
| --- | --- | --- | --- | --- | --- | --- | --- |
|  | SCZ (n=54) | BD (n=40) | Controls  (n=80) | Statistic, df, p-value | AA (n=84) | AC + CC  (n=90) | Statistic, df, p-value |
| Age (SD) | 37.0 (11.1) | 40.1 (12.3) | 39.1 (13.2) | F=0.80, df=2, p=0.45 | 39.9 (13.1) | 37.6 (11.6) | t=1.26, df=165.58, p=0.21 |
| IQ (SD) | 102.8 (13.1) | 110.9 (12.7) | 115.0 (10.8) | F=16.75, df=2, p < 0.001^a^ | 109.3 (13.7) | 111.2 (12.4) | t=-0.95, df=172, p=0.34 |
| IQ z-scores (SD) | -1.5 (1.6) | -0.3 (1.1) | 0.0 (0.9) | F=24.55, df=2, p < 0.001^a^ | -0.7 (1.4) | -0.4 (1.3) | t=-1.18, df=172, p=0.24 |
| CPZ – equivalent antipsychotics dose (SD) | 566.5 (623.9) | 115.7 (198.9) | n/a | t=4.98, df=66.84, p < 0.001^a^ | 449.5 (604.8) | 315.1 (464.5) | t=1.22, df=92, p=0.23 |
| Years of Education (SD) | 13.9 (2.3) | 14.9 (3.2) | 14.9 (2.6) | F=2.75, df=2, p=0.07 | 14.3 (2.5) | 14.8 (2.8) | t=-1.39, df=172, p=0.17 |
| Gender (Male / Female) | 42/12 | 16/24 | 34/46 | χ^2^=19.56, df=2, p < 0.001^a^ | 45/39 | 47/43 | χ^2^=0.0007, df=1, p=0.98 |
| Ethnicity (Caucasian / Black Caribbean /  Black African / Mixed African-Caucasian) | 47/5/1/1 | 38/1/1/0 | 77/1/1/1 | χ^2^=6.70, df=6, p=0.35 | 74/6/3/1 | 88/1/0/1 | χ^2^=7.58, df=3, p=0.06 |
| Handedness (Right / Left / Mixed) | 51/2/1 | 35/3/2 | 76/4/0 | χ^2^=4.70, df=4, p=0.32 | 75/8/1 | 87/1/2 | χ^2^=6.47, df=2, p=0.04 |

*^a^ Statistically significant at p < 0.001.*

*^b^ IQ was assessed using the WASI-II (Wechsler Adult Intelligence Scale-II)*^2^*, the WASI-III (Wechsler Adult Intelligence Scale-III)*^3^*, the WAIS-R (Wechsler Adult Intelligence Scale-Revised)*^4^*, the WASI-FSIQ-4(Wechsler Abbreviated Scale of Intelligence – Full Scale IQ)*^5^*, the Quick Test*^6^ *or the NART (National Adult Reading Test)*^7^*. The proportion of subjects assessed with each tool was matched between diagnostic or genotype groups and ANOVA was performed with standardized scores (z-score) based on the mean and standard deviation of the controls group for each tool.*

*^c^ Mean and standard deviation of non-standardized IQ data shown for easier interpretation.
n/a, not applicable; BD, bipolar disorder; SCZ, schizophrenia; AA, adenine-adenine; AC, adenine-cytosine; CC – cytosine-cytosine; CPZ, chlorpromazine; SD, standard deviation; df, degrees of freedom.*

**References**

1 R Core Team. *R: A language and environment for statistical computing*. R Foundation for Statistical Computing, 2016 (https://www.R-project.org/).

2 Wechsler D. Wechsler Abbreviated Scale of Intelligence – Second Edition Manual. 2011.

3 Wechsler D. Wechsler Adult Intelligence Scale – Third Edition Manual. 1997.

4 Wechsler D. WAIS-R manual: Wechsler adult intelligence scale – revised. 1981.

5 Wechsler D. Wechsler Abbreviated Scale of Intelligence. 1999.

6 Ammons R, Ammons C. The Quick test. 1962.

7 Nelson H, Willison J. The revised national adult reading test – test manual. 1991.
