# Supplementary material for: The impact of psychosis genome-wide associated ZNF804A variation on verbal fluency connectivity
Source: J Psychiatr Res. 2018 Mar;98:17–21. doi: 10.1016/j.jpsychires.2017.12.005 (PMC5793999; doi:10.1016/j.jpsychires.2017.12.005)
Supplement: Supplement 3 [file mmc3.docx]

***SUPPLEMENT 3***

**Supplementary Table 2 –** Regions showing an effect of *task on regional activation*. All inferences presented in this table correspond to statistically significant results (corrected for whole-brain multiple comparisons, voxel-level FWE, p < 0.05). C*luster size (k) is given only for the peak of each cluster.*

| ***Effects of task on regional activation*** | | | |
| --- | --- | --- | --- |
| **Contrasts** | **Regions** | **Coordinates (x y z)** | **Z-score (Z), voxel-wise FWE corrected p-value (p), cluster size (k)** |
| Verbal fluency > word repetition | L Precentral gyrus/ Inferior frontal gyrus, pars opercularis | -44 4 34 | Z=6.5x10^4^, p<0.001, k=15749 |
|  |  | 46 10 24 | Z=4.48, p=0.017, k=23 |
|  | L Insula | -32 22 4 | Z=6.5x10^4^, p<0.001 |
|  | R Insula | 34 22 0 | Z=6.5x10^4^, p<0.001 |
|  | R Caudate nucleus/ Septal nuclei | 20 -2 24 | Z=6.5x10^4^, p<0.001 |
|  |  | 24 -26 26 | Z=6.55x10^4^, p<0.001 |
|  | L Thalamus | -14 -4 14 | Z=6.55x10^4^, p<0.001 |
|  |  | -2 -24 12 | Z=6.55x10^4^, p<0.001 |
|  | L Inferior parietal gyrus | -40 -42 40 | Z=6.55x10^4^, p<0.001 |
|  |  | -26 -66 42 | Z=7.40, p<0.001 |
|  | L Middle cingulate gyrus | -4 20 32 | Z=6.55x10^4^, p<0.001 |
|  | R Middle cingulate gyrus | 8 18 32 | Z=7.79, p<0.001 |
|  |  | 8 24 30 | Z=7.63, p<0.001 |
|  | L Posterior cingulate gyrus | -18 -42 14 | Z=7.57, p<0.001 |
|  | L Precuneus | -28 -60 4 | Z=7.37, p<0.001 |
|  | R Fusiform gyrus | 34 -46 2 | Z=7.24, p<0.001 |
|  | Anterior Cerebellum (Vermis III) | -2 -34 0 | Z=7.12, p<0.001 |
|  | L Middle frontal gyrus | -34 50 22 | Z=5.00, p=0.002, k=43 |
|  | R Middle temporal gyrus | 50 -28 -10 | Z=5.47, p=0.017, k=15 |
| Word repetition > verbal fluency | R Middle cingulate gyrus | 6 -50 36 | Z=6.55x10^4^, p<0.001, k=5899 |
|  | R Angular gyrus | 48 -66 36 | Z=6.55x10^4^, p<0.001, k=1440 |
|  |  | 48 -58 28 | Z=6.55x10^4^, p<0.001 |
|  | R Middle temporal gyrus | 54 -60 12 | Z=6.55x10^4^, p<0.001 |
|  | R Middle occipital gyrus | 42 -78 18 | Z=6.12, p<0.001 |
|  |  | 34 -82 20 | Z=5.74, p<0.001 |
|  |  | 30 -86 18 | Z=5.72, p<0.001 |
|  |  | 38 -72 28 | Z=5.34, p<0.001 |
|  | L Angular gyrus | -50 -62 24 | Z=6.55x10^4^, p<0.001, k=939 |
|  |  | -50 -64 38 | Z=6.55x10^4^, p<0.001 |
|  |  | -46 -68 40 | Z=6.55x10^4^, p<0.001 |
|  | L Middle temporal gyrus | -52 -66 8 | Z=5.75, p<0.001 |
|  | L Middle occipital gyrus | -44 -78 8 | Z=4.70, p=0.007 |
|  |  | -26 -94 4 | Z=6.49, p<0.001, k=108 |
|  |  | -24 -96 4 | Z=6.45, p<0.001 |
|  |  | -20 -94 0 | Z=5.97, p<0.001 |
|  |  | -30 -92 8 | Z=5.75, p<0.001 |
|  |  | -40 -80 10 | Z=4.48, p=0.017 |
|  | L Anterior cingulate gyrus | -6 38 0 | Z=6.55x10^4^, p<0.001, k=440 |
|  | R Anterior cingulate gyrus | 8 36 0 | Z=6.55x10^4^, p<0.001 |
|  | R Superior frontal gyrus, orbital part | 22 34 -12 | Z=6.77, p<0.001 |
|  |  | 18 24 -12 | Z=5.24, p=0.001 |
|  | R Anterior cingulate gyrus | 4 50 12 | Z=6.55x10^4^, p<0.001, k=744 |
|  | R Superior frontal gyrus, medial part | 4 56 10 | Z=6.55x10^4^, p<0.001 |
|  | R Cuneus | 20 -98 8 | Z=7.80, p<0.001, k=208 |
|  | R Lingual gyrus | 18 -86 -6 | Z=7.42, p<0.001 |
|  |  | 22 -82 -8 | Z=6.37, p<0.001 |
|  | R Calcarine sulcus (occipital gyrus) | 18 -96 4 | Z=7.56, p<0.001 |
|  | R Fusiform gyrus | 26 -38 -18 | Z=7.72, p<0.001, k=154 |
|  | L Superior temporal gyrus | -40 -16 -2 | Z=7.66, p<0.001, k=1388 |
|  | L Rolandic operculum | -38 -20 18 | Z=7.45, p<0.001 |
|  | R Insula | 38 -14 12 | Z=7.49, p<0.001, k=1507 |
|  | R Putamen | 36 -14 2 | Z=7.36, p<0.001 |
|  | R Rolandic operculum | 52 -26 18 | Z=6.69, p<0.001 |
|  |  | 50 -20 14 | Z=6.48, p<0.001 |
|  | L Caudate gyrus/ Septal nuclei | -8 8 -12 | Z=5.21, p=0.001, k=33 |
|  |  | -4 8 -10 | Z=5.15, p=0.001 |
|  | R Thalamus | 14 -26 2 | Z=5.04, p=0.001, k=28 |
|  | R Olfactory gyrus | 4 8 -8 | Z=4.38, p=0.025, k=1 |

*R, right; L, left.*
