# Supplementary material for: The impact of psychosis genome-wide associated ZNF804A variation on verbal fluency connectivity
Source: J Psychiatr Res. 2018 Mar;98:17–21. doi: 10.1016/j.jpsychires.2017.12.005 (PMC5793999; doi:10.1016/j.jpsychires.2017.12.005)
Supplement: Supplement 4 [file mmc4.docx]

***SUPPLEMENT 4***

**Supplementary Table 3 –** Associations between rs1344706 genotype and ZNF804A transcription in different brain regions of the healthy brain

| **exprID** | **chr** | **start** | **stop** | **tID** | **CRBL** | **FCTX** | **HIPP** | **MEDU** | **OCTX** | **PUTM** | **SNIG** | **TCTX** | **THAL** | **WHMT** |
| --- | --- | --- | --- | --- | --- | --- | --- | --- | --- | --- | --- | --- | --- | --- |
| t2518889 | chr2 | 185463093 | 185970174 | 2518889 | 0.8 | 0.092 | 0.52 | 0.24 | 0.14 | 0.19 | 0.15 | 0.48 | 0.76 | 0.96 |
| 2518890 | chr2 | 185463126 | 185463171 | 2518889 | 0.66 | 0.95 | 0.032^a^ | 0.84 | 0.84 | 0.097 | 0.097 | 0.052 | 0.62 | 0.37 |
| 2518891 | chr2 | 185463193 | 185463277 | 2518889 | 0.89 | 0.084 | 0.41 | 0.95 | 0.99 | 0.41 | 0.78 | 0.47 | 0.98 | 0.18 |
| 2518892 | chr2 | 185463402 | 185463560 | 2518889 | 0.38 | 0.17 | 0.36 | 0.42 | 0.44 | 0.35 | 0.92 | 0.7 | 0.44 | 0.81 |
| 2518893 | chr2 | 185463699 | 185463781 | 2518889 | 0.92 | 0.57 | 0.83 | 0.23 | 0.74 | 0.97 | 0.28 | 0.53 | 0.45 | 0.92 |
| 2518918 | chr2 | 185731114 | 185731187 | 2518889 | 0.65 | 0.041 | 0.66 | 0.29 | 0.5 | 0.26 | 0.87 | 0.27 | 0.88 | 0.14 |
| 2518929 | chr2 | 185798405 | 185798434 | 2518889 | 0.14 | 0.8 | 0.59 | 0.49 | 0.14 | 0.23 | 0.79 | 0.64 | 0.89 | 0.32 |
| 2518930 | chr2 | 185800519 | 185800552 | 2518889 | 0.7 | 0.17 | 0.63 | 0.035^a^ | 0.38 | 0.16 | 0.083 | 0.57 | 0.59 | 0.4 |
| 2518931 | chr2 | 185800775 | 185801121 | 2518889 | 0.68 | 0.22 | 0.99 | 0.42 | 0.036^a^ | 0.35 | 0.14 | 0.6 | 0.49 | 0.25 |
| 2518932 | chr2 | 185801271 | 185802893 | 2518889 | 0.66 | 0.072 | 0.28 | 0.41 | 0.26 | 0.2 | 0.068 | 0.41 | 0.72 | 0.76 |
| 2518933 | chr2 | 185803086 | 185803238 | 2518889 | 0.38 | 0.27 | 0.3 | 0.43 | 0.11 | 0.059 | 0.62 | 0.21 | 0.59 | 0.082 |
| 2518935 | chr2 | 185803373 | 185803420 | 2518889 | 0.62 | 0.51 | 0.59 | 0.63 | 0.43 | 0.41 | 0.1 | 0.51 | 0.81 | 0.33 |
| 2518937 | chr2 | 185803561 | 185803594 | 2518889 | 0.76 | 0.69 | 0.31 | 0.43 | 0.34 | 0.11 | 0.067 | 0.54 | 0.92 | 0.48 |
| 2518940 | chr2 | 185803794 | 185804137 | 2518889 | 0.84 | 0.21 | 0.45 | 0.079 | 0.053 | 0.16 | 0.9 | 0.58 | 0.55 | 0.78 |

*Association is represented by the value of its significance (p-value).*

^a^ *Statistically significant (uncorrected p>0.05)*

*rsid, SNP identification; exprID, identification of the array’s probe used for transcript abundancy determination; aveALL, average of all brain areas; CRBL, Cerebellum; FCTX, Frontal cortex; HIPP, Hippocampus; MEDU, Medulla oblongata; OCTX, Occipital cortex; PUTM, Putamen; SNIG, substantia nigra; TCTX, temporal cortex; THAL, Thalamus; WHMT, White Matter.*
