# Supplementary material for: The impact of psychosis genome-wide associated ZNF804A variation on verbal fluency connectivity
Source: J Psychiatr Res. 2018 Mar;98:17–21. doi: 10.1016/j.jpsychires.2017.12.005 (PMC5793999; doi:10.1016/j.jpsychires.2017.12.005)
Supplement: Supplement 5 [file mmc5.docx]

***SUPPLEMENT 5***

**Supplementary Table 4 –** Detailed distribution of ZNF804A mRNA in the human brain, according to the Allen Brain Atlas. Relatively ZNF804A-enriched areas (mean normalized Z-score >1) were: the bed nucleus of stria terminalis, the nucleus of the diagonal band, the olfactory tubercle, the septal nuclei, the claustrum, the medial habenula, the hippocampal formation, the hypothalamus and the oculomotor and cochlear nuclei.

| **Brain region** | **Brain structure** | **Z-score** |
| --- | --- | --- |
| amygdala | amygdalohippocampal transition zone | 0,47 |
|  | basolateral nucleus | -0,42 |
|  | basomedial nucleus | 0,34 |
|  | central nucleus | 0,90 |
|  | cortico-medial group | 0,80 |
|  | lateral nucleus | -0,63 |
| basal forebrain | basal nucleus of meynert | 0,53 |
|  | bed nucleus of stria terminalis, | 1,34 |
|  | nucleus of the diagonal band, horizontal | 2,37 |
|  | nucleus of the diagonal band, vertical | 2,12 |
|  | olfactory tubercle | 1,03 |
|  | septal nuclei | 1,81 |
|  | substantia innominata | 0,77 |
| basal part of pons | pontine nuclei | 0,36 |
| cerebellar cortex | Crus I, lateral hemisphere | -0,32 |
|  | Crus I, paravermis | -0,02 |
|  | Crus II, lateral hemisphere | -0,27 |
|  | Crus II, paravermis | -0,16 |
|  | I-II | 0,37 |
|  | III | -0,14 |
|  | III, lateral hemisphere | 0,76 |
|  | III, paravermis | 0,33 |
|  | IV | -0,12 |
|  | IV, lateral hemisphere | 0,45 |
|  | IV, paravermis | 0,24 |
|  | IX | -0,36 |
|  | IX, lateral hemisphere | 0,03 |
|  | IX, paravermis | -0,52 |
|  | V | 0,15 |
|  | V, lateral hemisphere | 0,19 |
|  | V, paravermis | 0,35 |
|  | VI | 0,55 |
|  | VI, lateral hemisphere | 0,29 |
|  | VI, , paravermis | 0,02 |
|  | VIIAf | -0,05 |
|  | VIIAt | -0,72 |
|  | VIIB | 0,10 |
|  | VIIB, lateral hemisphere | -0,15 |
|  | VIIB, paravermis | -0,01 |
|  | VIIIA | -0,06 |
|  | VIIIA, lateral hemisphere | -0,25 |
|  | VIIIA, paravermis | -0,31 |
|  | VIIIB | -0,27 |
|  | VIIIB, lateral hemisphere | 0,13 |
|  | VIIIB, paravermis | -0,47 |
|  | X | 0,72 |
|  | X, lateral hemisphere | 0,47 |
|  | X, paravermis | 0,08 |
| cerebellar nuclei | dentate nucleus | 0,34 |
|  | fastigial nucleus | 0,04 |
|  | globose nucleus | -1,24 |
| cingulate gyrus | cingulate gyrus, frontal part, inferior bank | -0,21 |
|  | cingulate gyrus, frontal part, superior bank | -0,06 |
|  | cingulate gyrus, parietal part, inferior bank | 0,06 |
|  | cingulate gyrus, parietal part, superior ban | -0,14 |
|  | cingulate gyrus, retrosplenial part, inferior | -0,05 |
|  | cingulate gyrus, retrosplenial part, superior | -0,09 |
|  | subcallosal cingulate gyrus | -0,37 |
| claustrum | claustrum | 2,25 |
| dorsal thalamus | anterior group of nuclei | -0,72 |
|  | caudal group of intralaminar nuclei | -1,29 |
|  | dorsal lateral geniculate nucleus | -0,11 |
|  | lateral group of nuclei, dorsal division | -1,32 |
|  | lateral group of nuclei, ventral division | -0,86 |
|  | medial geniculate complex | -0,86 |
|  | medial group of nuclei | -0,40 |
|  | posterior group of nuclei | -0,36 |
|  | rostral group of intralaminar nuclei | -0,31 |
| epithalamus | lateral habenular nucleus | -0,71 |
|  | medial habenular nucleus | 2,44 |
|  | paraventricular nuclei, of thalamus | -1,39 |
|  | pineal gland | -1,98 |
| frontal lobe | anterior orbital gyrus | -0,55 |
|  | frontal operculum | -0,06 |
|  | frontal pole, inferior aspect | -0,62 |
|  | frontal pole, medial aspect | 0,88 |
|  | gyrus rectus | -0,07 |
|  | inferior frontal gyrus, opercular part | -0,10 |
|  | inferior frontal gyrus, orbital part | -0,11 |
|  | inferior frontal gyrus, triangular part | 0,00 |
|  | inferior rostral gyrus | -0,05 |
|  | lateral orbital gyrus | 0,01 |
|  | medial orbital gyrus | -0,08 |
|  | middle frontal gyrus, inferior bank of gyrus | 0,03 |
|  | middle frontal gyrus, superior bank of gyrus | -0,16 |
|  | paracentral lobule, anterior part | 0,15 |
|  | paracentral lobule, anterior part, inferior | 0,03 |
|  | paracentral lobule, anterior part, superior | -0,13 |
|  | parolfactory gyri | 0,08 |
|  | posterior orbital gyrus | 0,06 |
|  | precentral gyrus, bank of the central sulcus | 0,25 |
|  | precentral gyrus, bank of the precentral sul | 0,26 |
|  | precentral gyrus, inferior lateral aspect of | 0,20 |
|  | precentral gyrus, superior lateral aspect of | 0,11 |
|  | superior frontal gyrus, lateral bank of gyru | -0,15 |
|  | superior frontal gyrus, medial bank of gyrus | -0,07 |
|  | superior rostral gyrus | -0,24 |
| globus pallidus | globus pallidus, external segment | -0,84 |
|  | globus pallidus, internal segment | -0,74 |
| hippocampal formation | CA1 field | 1,42 |
|  | CA2 field | 2,10 |
|  | CA3 field | 0,72 |
|  | CA4 field | 0,17 |
|  | dentate gyrus | 1,63 |
|  | subiculum | 0,03 |
| hypothalamus | anterior hypothalamic area | 0,50 |
|  | arcuate nucleus of the hypothalamus | 0,26 |
|  | dorsomedial hypothalamic nucleus | 1,16 |
|  | lateral hypothalamic area, anterior region | 0,06 |
|  | lateral hypothalamic area, mammillary region | 0,36 |
|  | lateral hypothalamic area, tuberal region | 1,17 |
|  | lateral mammillary nucleus | 1,92 |
|  | lateral tuberal nucleus | -0,34 |
|  | mammillary body | -1,44 |
|  | medial mammillary nucleus | -0,85 |
|  | pallidohypothalamic nucleus | 0,62 |
|  | paraventricular nucleus of the hypothalamus | 1,21 |
|  | perifornical nucleus | 1,55 |
|  | posterior hypothalamic area | 0,77 |
|  | preoptic region | 0,01 |
|  | supramammillary nucleus | 1,65 |
|  | supraoptic nucleus | 1,57 |
|  | tuberomammillary nucleus | 1,33 |
|  | ventromedial hypothalamic nucleus | 0,61 |
| insula | long insular gyri | -0,18 |
|  | short insular gyri | 0,08 |
| mesencephalon | central gray substance of midbrain | -0,23 |
|  | cuneiform nucleus | 0,49 |
|  | inferior colliculus | 0,60 |
|  | midbrain raphe nuclei | 0,29 |
|  | oculomotor nuclear complex | 1,44 |
|  | pretectal region | -0,84 |
|  | red nucleus | -1,37 |
|  | subcuneiform nucleus | -0,06 |
|  | substantia nigra, pars compacta | -0,95 |
|  | substantia nigra, pars reticulata | -1,44 |
|  | superior colliculus | -0,55 |
|  | trochlear nucleus | -0,35 |
|  | ventral tegmental area | -1,04 |
| myelencephalon | arcuate nucleus of medulla | 0,19 |
|  | central glial substance | -1,75 |
|  | central medullary reticular group | 0,06 |
|  | cochlear nuclei | 1,25 |
|  | cuneate nucleus | -0,30 |
|  | dorsal motor nucleus of the vagus | -0,65 |
|  | gigantocellular group | 0,62 |
|  | gracile nucleus | -1,19 |
|  | hypoglossal nucleus | 0,30 |
|  | inferior olivary complex | 0,35 |
|  | lateral medullary reticular group | 0,17 |
|  | raphe nuclei of medulla | -0,23 |
|  | spinal trigeminal nucleus | 0,34 |
|  | vestibular nuclei | -0,42 |
| occipital lobe | cuneus, peristriate | 0,00 |
|  | cuneus, striate | -0,14 |
|  | inferior occipital gyrus, inferior bank | -0,46 |
|  | inferior occipital gyrus, superior bank | -0,37 |
|  | lingual gyrus, peristriate | -0,27 |
|  | lingual gyrus, striate | -0,44 |
|  | occipital pole, inferior aspect | 0,43 |
|  | occipital pole, lateral aspect | 0,06 |
|  | occipital pole, superior aspect | -0,03 |
|  | occipito-temporal gyrus, inferior bank | -0,06 |
|  | occipito-temporal gyrus, superior bank | -0,54 |
|  | superior occipital gyrus, inferior bank | -0,36 |
|  | superior occipital gyrus, superior bank | -0,07 |
| parahippocampal gyrus | parahippocampal gyrus | -0,44 |
|  | parahippocampal gyrus, lateral bank | -0,17 |
| parietal lobe | angular gyrus, inferior bank | -0,22 |
|  | angular gyrus, superior bank | 0,00 |
|  | postcentral gyrus, central | 0,44 |
|  | postcentral gyrus, posterior | -0,18 |
|  | postcentral gyrus, inferior lateral | 0,00 |
|  | postcentral gyrus, superior lateral | -0,04 |
|  | precuneus, inferior lateral bank | -0,24 |
|  | precuneus, superior lateral bank | -0,19 |
|  | superior parietal lobule, inferior bank | 0,06 |
|  | superior parietal lobule, superior bank | -0,07 |
|  | supramarginal gyrus, inferior bank | 0,00 |
|  | supramarginal gyrus, superior bank | -0,13 |
| piriform cortex | piriform cortex | 0,14 |
| pontine tegmentum | abducens nucleus | 0,26 |
|  | central gray of the pons | 0,24 |
|  | facial motor nucleus | 0,89 |
|  | lateral parabrachial nucleus | 0,28 |
|  | locus ceruleus | -0,27 |
|  | medial parabrachial nucleus | 0,54 |
|  | motor nucleus of trigeminal nerve | 0,92 |
|  | nucleus subceruleus | 0,10 |
|  | pontine raphe nucleus | 0,06 |
|  | pontine raphe nucleus | -0,06 |
|  | pontine reticular formation | 0,35 |
|  | principal sensory nucleus of trigeminal nerve | 0,67 |
|  | superior olivary complex | -0,66 |
| striatum | body of caudate nucleus | -0,31 |
|  | head of caudate nucleus | -0,44 |
|  | nucleus accumbens | 0,05 |
|  | putamen | -0,21 |
|  | tail of caudate nucleus | 0,16 |
| subthalamus | subthalamic nucleus | -1,61 |
| Choroid plexus | choroid plexus of the lateral ventricle | -2,00 |
|  | fusiform gyrus | -0,15 |
|  | fusiform gyrus, lateral bank | -0,21 |
|  | Heschl's gyrus | -0,23 |
|  | inferior temporal gyrus, bank of mts | -0,15 |
|  | inferior temporal gyrus, bank of the its | -0,15 |
|  | inferior temporal gyrus, lateral bank | -0,24 |
|  | middle temporal gyrus, inferior bank | -0,23 |
|  | middle temporal gyrus, superior bank | -0,13 |
|  | planum polare | -0,24 |
|  | planum temporale | 0,23 |
|  | superior temporal gyrus, inferior bank | 0,04 |
|  | superior temporal gyrus, lateral bank | -0,03 |
|  | temporal pole, inferior aspect | 0,17 |
|  | temporal pole, medial aspect | 0,38 |
|  | temporal pole, superior aspect | -0,22 |
|  | transverse gyri | 0,52 |
| ventral thalamus | reticular nucleus of thalamus | 0,73 |
|  | zona incerta | -0,18 |
| white matter | cingulum bundle | -1,15 |
|  | corpus callosum | -1,92 |

**Supplementary Figure 1:** Brain region-based distribution of ZNF804A mRNA in the human brain.

*
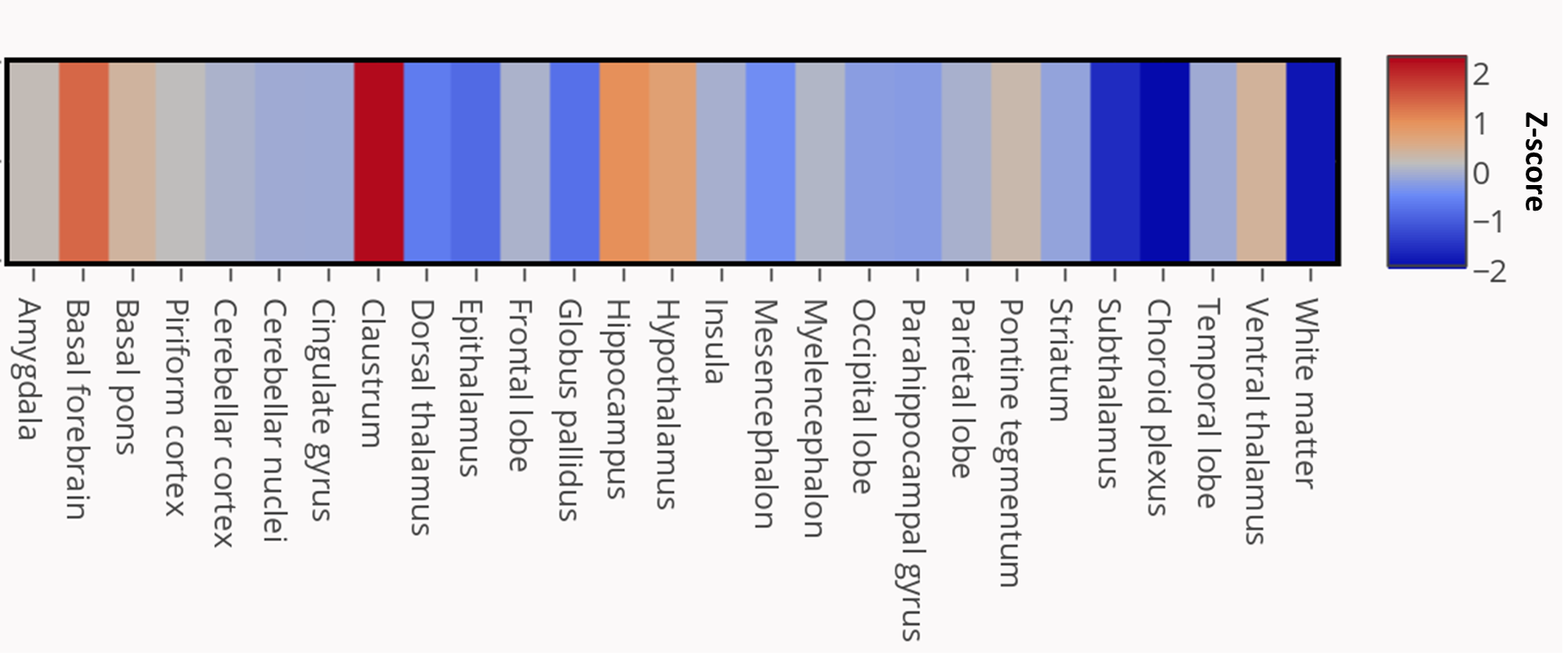
*

**Supplementary Figure 2**: Brain structure-distribution of ZNF804A mRNA in the human brain.

*
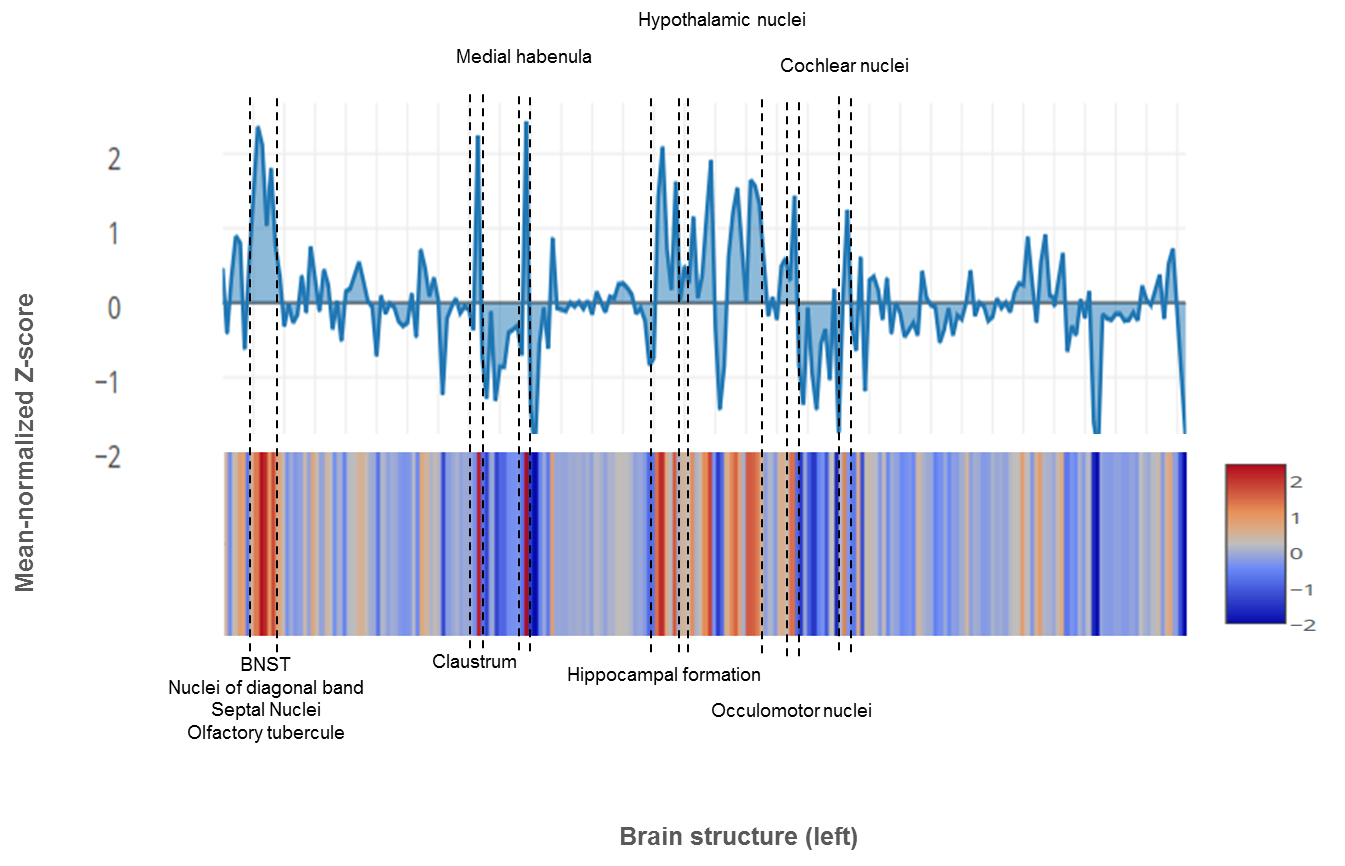
*
